# Silicone Rubber Based Highly Sensitive Fiber-Optic Fabry–Perot Interferometric Gas Pressure Sensor

**DOI:** 10.3390/s20174927

**Published:** 2020-08-31

**Authors:** Xin Cheng, Jitendra Narayan Dash, Dinusha Serandi Gunawardena, Lin Htein, Hwa-Yaw Tam

**Affiliations:** Department of Electrical Engineering, Photonics Research Centre, The Hong Kong Polytechnic University, Kowloon, Hong Kong 999077, China; eechengx@polyu.edu.hk (X.C.); dinusha.gunawardena@polyu.edu.hk (D.S.G.); hwa-yaw.tam@polyu.edu.hk (H.-Y.T.)

**Keywords:** optical fiber, Fabry–Perot interferometer (FPI), gas pressure sensor, silicone rubber

## Abstract

A simple, compact, and highly sensitive gas pressure sensor based on a Fabry–Perot interferometer (FPI) with a silicone rubber (SR) diaphragm is demonstrated. The SR diaphragm is fabricated on the tip of a silica tube using capillary action followed by spin coating. This process ensures uniformity of its inner surface along with reproducibility. A segment of single mode fiber (SMF) inserted into this tube forms the FPI which produces an interference pattern with good contrast. The sensor exhibits a high gas pressure sensitivity of −0.68 nm/kPa along with a low temperature cross-sensitivity of ≈ 1.1 kPa/°C.

## 1. Introduction

Fiber-optic Fabry–Perot interferometric pressure sensors have been attracting the attention in a wide range of fields such as biomedicine, healthcare, civil engineering, petroleum, automotive, and aerospace industries for their high sensitivity and immunity to electromagnetic interference [1,2,3]. Furthermore, these types of interferometers are compact and easy to be used. Some Fabry–Perot interferometer (FPI) sensors utilize change in the cavity length while others utilize the variation in refractive index (RI) of the cavity medium to detect pressure [4,5,6,7]. However, the former is more convenient for practical applications. Furthermore, single cavity-based interferometers are preferred due to the ease of resolving the corresponding interference pattern.

Many FPI pressure sensors based on the detection of cavity-length change have been reported [1,4,8,9]. For example, W.P. Chen et al. have reported a multi-mode fiber (MMF) based gas pressure sensor with a high sensitivity of −40.94 nm/MPa by using a large core area [1]. T.W. Fu et al. have reported a liquid level based pressure measurement system that employed a hollow core fiber coated with NOA65 [9]. M. Yao et al. have reported one of the smallest FPI sensors using µ-Printed SU-8 polymer, coated on a fiber tip which has a sensitivity of 4.29 nm/MPa [4]. Most of the aforementioned sensors show relatively low sensitivities and are unsuitable for low pressure measurements. Although some of these sensors exhibit high sensitivities, they involve complex fabrication processes. For example, 3D micro printing on the tip of the fiber to make a FP cavity and controlling the thickness of the reflecting surface of the cavity involves complex processes [4]. Furthermore, laser micromachining requires complex optical set ups for fabrication of side opened probes [8]. For human physiological measurements, the required pressure typically varies from 0 to 20 kPa and in some scenarios it ranges from 0 to 1 kPa [3]. Pressure sensors suitable for this particular range have been reported by G.C. Hill et al. in which 3D lithographic technique was used to fabricate an FPI with SU-8 which involved a complex fabrication process [10]. In addition, graphene has also been utilized as a diaphragm to achieve a much higher pressure sensitivity of 39.4 nm/kPa [11]. Although graphene based FPIs show better sensitivities even in a low pressure range, the fabrication process of graphene is complicated and expensive. Additionally, thinner graphene-based diaphragms are extremely fragile because of vacancy defects [12]. Therefore, a gas pressure sensor involving a simple and low-cost fabrication procedure along with better performance needs to be explored.

In this study, we propose and experimentally demonstrate a novel fiber based FPI sensor for gas pressure measurement with high sensitivity and low temperature cross-sensitivity. The fabrication of the sensor involves a simple procedure with good repeatability. The sensor consists of a silica capillary coated with silicone rubber (SR). The end-face of a single mode fiber (SMF) inserted into the capillary acts as one of the reflecting surfaces while the silicone rubber acts as the second reflecting surface. Due to its much lower Young’s modulus, silicone rubber is extremely soft and the diaphragm fabricated from this material is sensitive even when subjected to a low air pressure level, thereby resulting in a high sensitivity. Additionally, silicone rubber is resistant to strong alkalis and acids and possesses good biocompatibility as well [13]. Thus, the proposed highly sensitive FPI sensor with excellent repeatability can be considered as a key asset for pressure measurements in biomedical applications such as respiration monitors [14].

## 2. Sensing Principle and Fabrication

**Figure 1:**
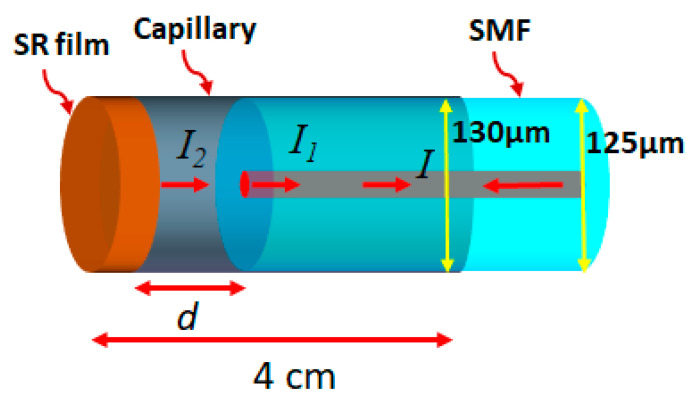
Schematic illustration Fabry–Perot interferometer (FPI) where d refers to the distance between silicone rubber (SR) film and tip of single mode fiber (SMF), *I_1_* and *I_2_* refer to the intensity of light reflected from the tip of SMF and the SR film, respectively, and *I* refers to the total intensity (*I_1_* + *I_2_*).

The schematic illustration of the proposed FPI for gas pressure sensing is shown in Figure 1. The FPI comprises of a section of silica capillary (diameter = 130 µm) coated with an SR film by spin coating method, and a section of SMF (diameter = 125 µm) inserted into this silica capillary. The inner surface of the SR film and the tip of SMF act as two reflecting surfaces for the resulting interference pattern.

### 2.1. FPI Sensing Principle

Light is coupled into the probe and gets reflected from the tip of SMF and the inner surface of the SR film. The opaque nature of the SR film avoids light being reflected from the outer surface. Therefore, the resulting spectrum can be described as a two-beam interference phenomenon. If *I_1_* and *I_2_* are the intensities of light reflected from the tip of SMF and the inner surface of the SR film, then the resulting interference pattern can be expressed as in Equation (1):

(1) $$I\;=\;I_{1}\;+\;I_{2}\;+\;2I_{1}I_{2}cos\left(\frac{4\pi nd}{\lambda }\right)$$

where *d* refers to the distance between the tip of SMF and inner surface of the film and *n* refers to the refractive index of the medium between the two reflecting surfaces. The FSR (free spectral range) of the spectrum can be expressed as in Equation (2):

(2) $$FSR\;=\;\frac{\lambda_{1}\lambda_{2}}{2dn}$$

where *λ_1_* and *λ_2_* refer to the wavelength of consecutive minima of the interference pattern. When air pressure is applied, the SR film moves inward owing to its flexible nature, thereby reducing the value of *d*. This leads to a blue shift of the interference pattern and by tracking the minima of the interference spectrum, the value of the applied pressure can be determined. 

### 2.2. Fabrication of Sensor

**Figure 2:**
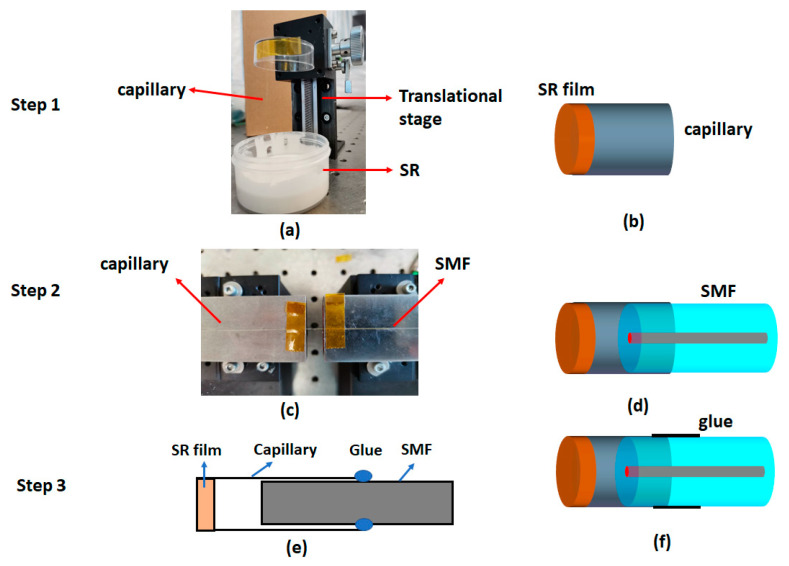
Step by step procedure for the fabrication of the FPI sensor. Step 1: dipping of capillary into the SR solution (**a**) and schematic of the capillary with SR film at its tip (**b**). Step 2: experimental set up for insertion of a SMF into the capillary (**c**) and schematic of the capillary with inserted SMF (**d**). Step 3: schematic of fixing of capillary with SMF using glue (**e**) and resulting probe (**f**), respectively.

The proposed FPI sensor was fabricated following the series of steps depicted in Figure 2. Firstly, a section of polyimide coated homemade silica capillary with an inner diameter of 130 µm was cleaved to a length of 4 cm. Then a mixture of SR (RHODORSIL RTV 573, Elkem Silicones, Leverkusen, Germany) and its catalyst was prepared at a base to catalyst weight ratio of 10:1. The mixture was left for 2 min and then the tip of the silica capillary was slightly dipped into the mixture with the aid of a translation stage as shown in Figure 2a. Due to the viscous nature of the mixture, the mixture gets attached and forms a ball like structure at the tip of the capillary. Simultaneously, droplets of this mixture were also placed on three glass slides. Afterwards, the capillary tip coated with SR was immediately fixed on a plastic stage vertically upwards, which in turn was placed on the rotating stage of the spin coating machine (CPN-KW-4A, Chemat, Shanghai, China). The duration of rotation was fixed at 30 s while the rotation speed was varied to optimize the surface quality of the film. Similarly, the glass slide was also placed on the spin coating machine and the rotation duration of the stage was kept same as that for capillary. After spin coating, the fiber as well as the glass slide were left at room temperature (25 °C) for 1 h so that the film was cured properly. The schematic of the SR film coated capillary is shown in Figure 2b. A segment of SMF was then inserted inside the SR coated capillary using a translation stage as shown in Figure 2c. During this process, the resulting interference pattern was monitored with an optical spectrum analyzer (OSA, Yokogawa AQ6375, resolution = 0.02 pm, Yokogawa, Tokyo, Japan). The schematic of the capillary with inserted SMF is shown in Figure 2d. SMF was fixed to the capillary using adhesive glue (LOCTITE 431, Henkel, Düsseldorf, Germany) at a point where interference spectrum with good visibility was observed. The schematic of this process as well as resulting probe is shown in Figure 2e,f, respectively. The glue also sealed the cavity ensuring it is air-tight.

**Figure 3:**
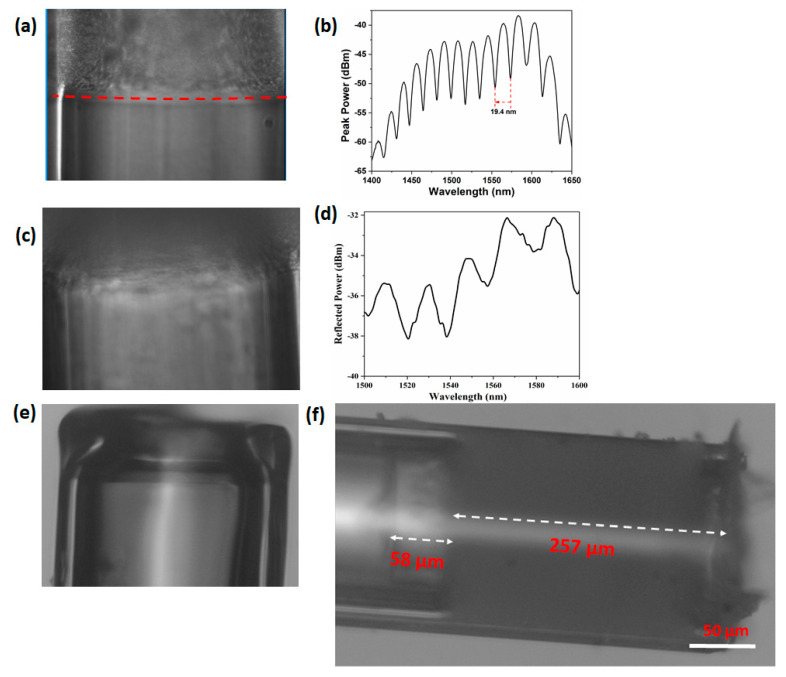
Optical microscopic images of the inner surface of the SR films formed at the tip of capillary after spin coating at rotational speeds of 4800 rpm (**a**) and the corresponding interference pattern (**b**); 3000 rpm (**c**) and the corresponding interference pattern (**d**). Tip of the capillary with no SR film after spin coating at a rotational speed of 5900 rpm (**e**). Optical microscopic image of fabricated probe corresponding to rotational speed of 4800 rpm (**f**).

**Figure 4:**
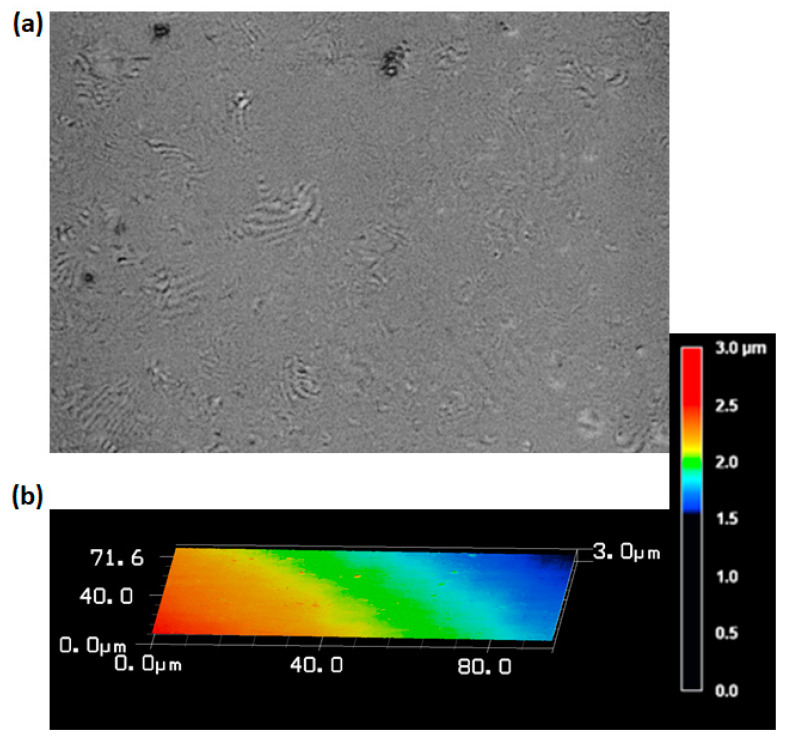
(**a**,**b**) The optical microscopic image and roughness profile of the SR film deposited on the glass plate, respectively.

The optical microscopic images of the inner surface of the SR film corresponding to rotational speeds of 4800, 3000, and 5900 rpm are shown in Figure 3a,c,e, respectively. At a rotational speed of 4800 rpm, the inner surface of the SR film appears to have a flat surface which can be verified from the commendable contrast of the corresponding interference pattern shown in Figure 3b. However, at a lower rotational speed of 3000 rpm, the inner surface of the SR film appears to be rough and curved as shown in Figure 3c and the corresponding interference pattern shows a diminished contrast in the reflected power as shown in Figure 3d. It should also be noted that such SR films with rough and curved inner surfaces can also be formed in the absence of the spin coating process. Further increment in the rotational speed complicates the uniform film formation process and hence, at a rotational speed of 5900 rpm, there is no visible film at the tip of the capillary as shown in Figure 3e. Therefore, 4800 rpm was verified as the optimized value of the rotational speed and the corresponding optical microscopic image of the probe is shown in Figure 3f where the thickness of the SR film is 257 µm and the cavity length is 58 µm and this length nearly agrees with that of the theoretical value (63 µm) calculated using Equation (2). Three probes with SR thicknesses of 251, 257, and 258 µm (standard deviation of 3.78 µm) were fabricated using the same optimized rotation speed. The roughness measurement of the SR film surface on the glass slide was also conducted using laser microscope (VK-X200, KEYENCE). The maximum variation in surface roughness of the samples are found to be 3, 3.3, and 2.5 µm with the standard deviation being 0.40 µm. The optical microscopic image of the film as well as roughness measurement for one of the samples is shown in Figure 4.

## 3. Experiments and Results

### 3.1. Experimental Procedure

**Figure 5:**
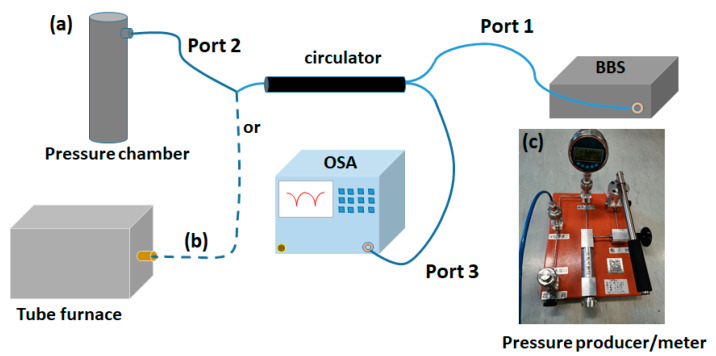
Experimental setup for pressure (**a**) and temperature (**b**) characterization. Pressure pump and gauge used during the pressure measurements (**c**).

The experimental setup used to detect and analyze the response of the FPI sensor to physical parameters such as applied gas pressure and temperature is displayed in Figure 5. Light from a broadband source (bandwidth of 100 nm) was coupled to one port of a circulator (6015-3-APC, Thorlabs Inc, New jersey, NJ, USA) and the probe was spliced to the second port of the circulator. The reflected light from the probe was collected by the third port connected to the OSA. The sensing probe was placed inside a gas pressure chamber as shown in Figure 5, in an attempt to investigate its pressure response, where the pressure inside the chamber was tuned gradually from 0 to 50 kPa at steps of 5 kPa. The applied pressure was calibrated using a pressure gauge (CONST 211) (see Figure 5b). Similarly, the temperature response of the probe was also explored by placing the sensing probe inside a tube furnace. Additionally, detection of the pressure response time was also carried out with the aid of an interrogator (Micro Optics, si255, Luna Lightwave, Atlanta, GA, USA) with a resolution of 1 pm and a scanning rate of 5 kHz.

### 3.2. Results and Discussion

**Figure 6:**
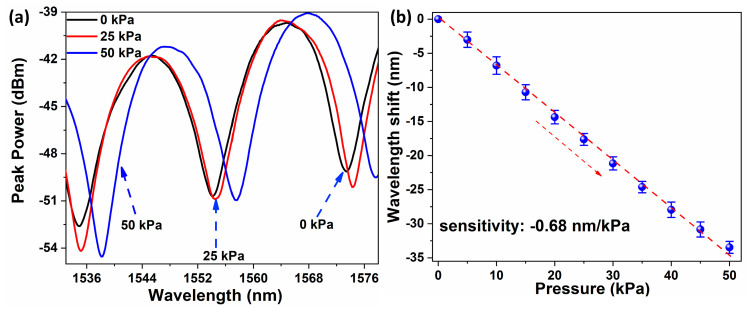
Response of spectral minima of the FPI sensor to changes in pressure (**a**) and the corresponding average shift in wavelength minima along with error bars (**b**).

Initially, the pressure response characteristics of the fabricated probe with applied gas pressure was investigated. The pressure in the chamber can be tuned using a pressure controller as shown in Figure 5c and a commercial pressure meter is used to monitor the pressure in the chamber. Figure 6a demonstrates the corresponding interference spectra when the pressure inside the chamber is increased from 0 to 50 KPa with a step size of 5 kPa. A blue shift in the spectral minima is apparent with increasing pressure in the chamber. The experiment was repeated for three cycles and the average response of the probe along with error bars are shown in Figure 6b. A good linearity in the pressure response with an R^2^ value of 0.998 and a sensitivity of −0.68 nm/kPa were obtained.

Different types of fiber based FPI gas pressure sensors along with their various structures and sensitivities are summarized in Table 1. Based on the table, the proposed probe shows a significantly higher sensitivity in low pressure regimes (kPa) compared to other FPI structures which are suitable for high pressure ranges (MPa). This relatively high pressure sensitivity in the proposed probe can be attributed to the much lower Young’s modulus of the SR film (≈0.9 MPa) compared to that of polyvinyl chloride (PVC), polydimethylsiloxane (PDMS), polycarbonate (PC), poly (methyl methacrylate) (PMMA), and polystyrene (PS) where the Young’s moduli are in the order of GPa [15,16]. Therefore, the soft SR film tends to compress easily resulting in a higher sensitivity. 

**Figure 7:**
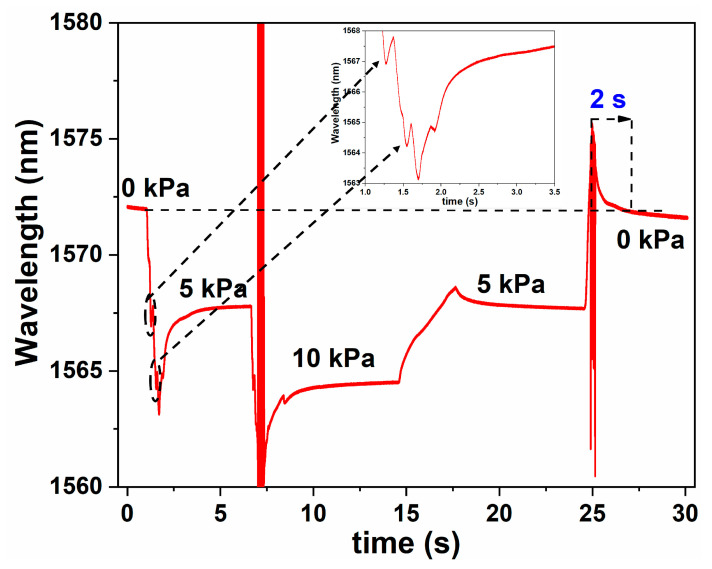
Response time of the probe to changes in applied gas pressure. The inset shows the expanded view of wavelength shift of the interreference minimum with increase in pressure.

The response time of the FPI sensor to changes in gas pressure was subsequently monitored using an interrogator. The wavelength of a particular spectral minimum was considered to analyze the dynamic response of the sensor. The pressure inside the chamber was increased from standard atmospheric pressure to 5 kPa at which point the pressure was held until the corresponding change in the wavelength of the spectral minimum stabilized. After wavelength stabilization, the pressure was raised again from 5 to 10 kPa. The response of the sensor is shown in Figure 7. The sensor shows a sudden leap in the wavelength for an increase in the gas pressure as can be seen from the inset of Figure 7 where the experimental response time is less than 2 s.

**Figure 8:**
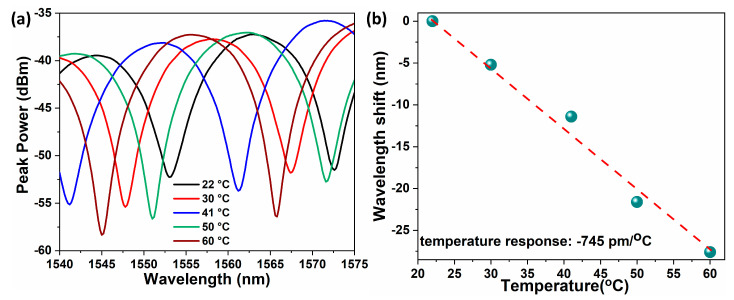
Response of the spectral minima of the FPI sensor to temperature variations (**a**) and corresponding wavelength shift of a specific spectral minimum (**b**).

The response of the probe for changes in ambient temperature was investigated by placing the probe in a tube furnace where the temperature was increased from 20 to 60 °C. With rising temperature, the spectral minimum shows a blue shift as can be seen from Figure 8a. The blue shift occurs due to the expansion of the SR film with increase in temperature (thermal expansion of silicone rubber is 250 × 10^−6^/K) thereby, reducing the cavity length. The shift in the wavelength with increasing temperature is plotted in Figure 8b which shows a linear variation with a temperature sensitivity of −0.745 nm/°C. Therefore, the error in pressure measurements resulting from cross temperature is found to be ≈ 1.1 kPa/°C which is much lower than that reported in previous research studies [21].

## 4. Conclusions

In summary, a highly sensitive gas pressure sensor using an FP based interferometer was demonstrated. An SR film at the tip of a silica capillary and the end-facet of an SMF inserted into this capillary form the cavity of the interferometer. The SR film was highly sensitive to the applied gas pressure owing to its much lower value of Young’s modulus. The sensor exhibited a gas pressure sensitivity of −0.68 nm/kPa along with a temperature sensitivity of −0.745 nm/°C that resulted in a low cross temperature sensitivity of 1.1 kPa/°C. Due to the simple fabrication method of the probe and excellent resistance of the SR film to acid, alkali, and UV light, the proposed interferometer can be considered as an ideal candidate for bio-sensing systems in testing surroundings.

## Figures and Tables

**Table 1:** Gas-pressure sensitivities of several fiber-optic FPI sensors.

FPI Structure	Sensitivity
FPI embedded in pressure fitting [17]	0.24 nm/MPa
Pendant polymer droplet-based FPI [2]	1.13 nm/MPa
FPI based on concave well on fiber end [18]	1.53 nm/MPa
FPI with open cavity [19]	2.46 nm/MPa
FPI based on dual capillaries [20]	4.15 nm/MPa
Hollow-core bandgap fiber with a side opened channel FPI [8]	4.24 nm/MPa
PVC-diaphragm based FPI [21]	65.5 nm/MPa
FPI sensor based on cavity length measurement [22]	0.42 nm/kPa
Silicone rubber-diaphragm based FPI	−680 nm/MPa
	(−0.68 nm/kPa)
